# Faster genetic mapping of complex traits in *C. elegans*

**DOI:** 10.17912/micropub.biology.001544

**Published:** 2025-04-23

**Authors:** Stefan Zdraljevic, Laura Walter-McNeill, Alex Lee, Joshua Bloom, Leonid Kruglyak

**Affiliations:** 1 Department of Human Genetics, University of California, Los Angeles, CA, USA; 2 Department of Biological Chemistry, University of California, Los Angeles, CA, USA; 3 Howard Hughes Medical Institute, Chevy Chase, MD, USA; 4 Massachusetts Institute of Technology, Cambridge, MA, USA; 5 Howard Hughes Medical Institute, Chevy Chase, MD

## Abstract

*Caenorhabditis elegans* is a tractable model system that enables the identification of genetic determinants that underlie phenotypic variation. Over the years, new approaches have been developed to lower the cost of and expedite genetic mapping in this model system. The *ce* X-QTL approach uses the *fog-2 ( q71 )* allele to create obligate outcrossing recombinant populations for selection and sequencing experiments. Here, we tested whether the *fog-2 ( q71 )* allele is essential to the *ce* X-QTL approach by comparing crosses between the N2 and XZ1516 strains using either *fog-2 ( q71 )* or *fog-2* RNAi knockdown to facilitate outcrossing. The genome-wide allele frequencies of the bulk recombinant populations derived from these two methods were largely similar. These results demonstrate that *fog-2* RNAi is a viable alternative for rapidly generating recombinant populations, allowing greater flexibility in experimental design.

**Figure 1. fog-2 RNAi is sufficient to construct bulk recombinant populations:**
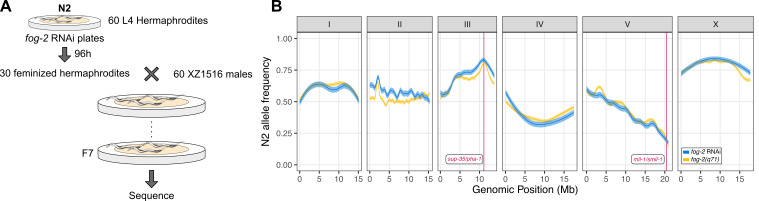
A) Experimental approach for the head-to-head comparison of crosses with *fog-2 ( q71 )* or *fog-2* RNAi. N2 L4s were transferred to *fog-2* RNAi plates and grown for 96 hours. Animals from the subsequent generation were considered feminized hermaphrodites if they displayed the stacked embryo phenotype. Thirty feminized N2 hermaphrodites were crossed to 60 XZ1516 males for 24 hours. Mated N2 animals were then transferred to 10 cm *fog-2* RNAi plates and allowed to lay F1 progeny. F2 L1 progeny from these plates were harvested and used to seed a fresh set of 10 cm *fog-2* RNAi plates. After seven generations of intercrossing, animals were harvested for sequencing. Crosses with the *fog-2 ( q71 )* were constructed in exactly the same way. B) N2 allele frequencies are shown on the y-axis for each of the six *C. elegans* chromosomes. Each tick on the x-axis corresponds to 5 Mb. Allele frequencies from crosses derived from the *fog-2 ( q71 )* strains are shown in yellow, and those from *fog-2* RNAi are shown in blue. The shaded areas represent the standard errors associated with the allele frequency estimates. Vertical red lines on chromosomes III and V show the positions of the *sup-35 / pha-1* and *mll-1/smll-1* TAs, respectively.

## Description

*Caenorhabditis elegans* has proven to be an exemplary model system to probe how genetic factors shape traits (Andersen & Rockman, 2022). The success of *C. elegans* as a model system for quantitative genetics can be attributed to its short generation time, relative ease of setting up crosses, well-annotated high-quality genome sequence, and a wide variety of molecular and population genetics tools (Brenner, 1974; C. elegans Sequencing Consortium, 1998; Cook et al., 2017).

A typical experiment to identify genetic factors underlying trait variation in the population involves constructing a panel of recombinant inbred lines derived from two phenotypically different parental strains (Andersen et al., 2015; Rockman & Kruglyak, 2009). However, constructing, genotyping, and phenotyping these panels is laborious and costly. We recently developed an alternative approach, *ce* X-QTL, that relies on incorporating the *fog-2 ( q71 )* loss of function allele into parental strains to make them obligate outcrossers (Burga et al., 2019; Schedl & Kimble, 1988). This allele enables construction of large pools of recombinant progeny that can be subjected to selection regimes to find the genetic basis of phenotypic variation in the population. To further expedite the mapping process, we asked whether the *fog-2 ( q71 )* allele is required for *ce* X-QTL.

To answer this question, we introduced the *fog-2 ( q71 )* allele into two genetically divergent *C. elegans* strains, XZ1516 and N2 . These two strains are incompatible at the *sup-35 / pha-1* and *mll-1/smll-1* toxin-antidote elements (TAs) (Ben-David et al., 2017; Zdraljevic et al., 2024), which served as genomic loci with strong and stereotypical effects on allele frequencies in recombinant populations. Our goal was to compare allele frequencies in a cross between XZ1516 ( *fog-2 ( q71 )* ) and N2 ( *fog-2 ( q71 )* ) to those from a cross between N2 and XZ1516 propagated on RNAi plates that knock down the expression of *fog-2* ( Figure 1A ) (Kamath et al., 2001; Timmons & Fire, 1998). Our rationale for this experiment was that temporary feminization of the parental lines may be sufficient to generate pools of recombinants that could be used for bulk selection experiments.

We sequenced the two resulting recombinant populations after seven generations of intercrossing on *fog-2* RNAi plates and calculated the N2 and XZ1516 allele frequencies across the genome. We observed the expected depletion of the N2 genotype at the *mll-1/smll-1* TA locus on chromosome V in both crosses ( Figure 1B ). Importantly, there wasn't a significant difference in allele frequencies at this locus between the two crosses ( *p* value= 0.89 at V:20469619). Similarly, we observed a depletion of the non-carrier XZ1516 genotype at the *sup-35 / pha-1* locus in both crosses, with only a minimal difference between the allele frequencies ( *p* value = 0.03 at III:11119227). The overall patterns of allele frequencies across the genome were very similar in both crosses, with both approaches detecting the same allele frequency distortions on chromosomes I, IV, and X, outside the known TAs.

Our results show that the *fog-2 ( q71 )* allele is not required for construction of bulk recombinant populations for *ce* X-QTL experiments. Instead, large recombinant populations can be quickly generated on *fog-2* RNAi to assess the genetic basis of trait variation. Once constructed, these populations can be taken off *fog-2* RNAi and maintained as hermaphrodite bulk populations, or, if desired, singled and amplified to construct recombinant inbred line panels. There are, however, limitations to using *fog-2* RNAi to construct bulk recombinant populations. It is known that there is substantial variation in RNAi sensitivity across the *C. elegans* population, and one would have to assess RNAi sensitivity of the parental lines prior to moving forward with crosses on *fog-2* RNAi plates (Chou et al., 2024; Paaby et al., 2015; Pollard & Rockman, 2013). Additionally, because RNAi is not fully penetrant in even the most sensitive *C. elegans* isolates, self progeny are expected to arise during the construction of *fog-2* RNAi-induced recombinant populations, which may reduce the mapping resolution at any given QTL by lowering the overall number of recombination events in the population. Given the limited number of recombination events per generation in *C. elegans* (Rockman & Kruglyak, 2009), we think that these are reasonable tradeoffs given the ease of construction of recombinant populations via *fog-2* RNAi.

## Methods

**Strains**

N2 and XZ1516 were acquired from the *Caenorhabditis elegans* Natural Diversity Resource (Cook et al., 2017). The *E. coli* strains OP50 and HB101 were acquired from the Caenorhabditis Genetics Center (CGC), which is funded by NIH Office of Research Infrastructure Programs (P40 OD010440). We previously described the introduction of the *fog-2(q71)* allele into XZ1516 to construct the QX2538 strain (Zdraljevic et al., 2024). We constructed QX2544[ *fog-2(q71[P17stop])* in N2] in the same manner.

**Strain maintenance**

Prior to growth on RNAi-expressing bacteria, all *C. elegans* strains were maintained at 20°C on modified nematode growth medium (NGMA), containing 1% agar and 0.7% agarose (Andersen et al., 2014) and seeded with OP50 *E. coli* . Spontaneous XZ1516 males were identified on 10 cm plates and used to establish male cross cultures.

**RNAi bacteria**

HT115 bacteria expressing *fog-2* RNAi in the L4440 plasmid background were acquired from Horizon Biosciences (Catalog#: RCE1182-202300280) (Rual et al., 2004; Timmons & Fire, 1998). Prior to induction, the fog-2 RNAi bacteria were streaked out to single colonies on LB + 100 µg/ml carbenicillin agar plates. Single colonies were grown overnight in 3 ml liquid LB + 100 µg/ml carbenicillin cultures. The following day, these cultures were diluted 1:1000 in LB + 100 µg/ml carbenicillin and grown for 16 hours. Finally, the expression of *fog-2* RNA was induced with 1 mM IPTG for four hours. 100 µl of 1000 µl of these cultures were used to seed 6 cm or 10 cm plates, respectively. RNAi plates were dried for two days at room temperature and then transferred to 4ºC. We only used RNAi plates that were seeded within seven days in all experiments.

**RNAi sensitivity**

To test the susceptibility of the strains to *fog-2* RNAi, we transferred ~10 N2 and XZ1516 L4 hermaphrodites to *fog-2* RNAi plates and allowed them to generate progeny. Once the progeny reach L4, we singled ~30 individuals to agar plates. The following day we counted the number of individuals that laid progeny. For both N2 and XZ1516, approximately 5% of the transferred individuals generated progeny, indicating that these strains are highly susceptible to RNAi.

**Cross construction**

Multiple 6 cm *fog-2* RNAi plates were seeded with five N2 L4s and incubated at 20ºC for 96 hours until adults from the next generation were present. Adults that expressed the *fog-2* -specific stacked oocyte phenotype were considered to be feminized (Qin & Hubbard, 2015). Thirty feminized N2 animals were crossed to 60 XZ1516 males on *fog-2* RNAi plates overnight and assessed for plugs the following day (Noble et al., 2015). Plugged N2 worms were then transferred to three 10 cm *fog-2* RNAi plates and allowed to lay progeny for 96 hours. 96 hours later, the resulting population was washed off these 10 cm plates with M9 media. The M9 containing the cross populations were transferred to a 15 ml conical tube and allowed to settle for 10 minutes. After this time ~75% of the supernatant in the conical tube was transferred to a new conical tube. The transferred media contained young larval F2 worms that were then titered to accurately seed the next generation. Approximately 15,000 young larvae were used to seed the subsequent generation with approximately 1000-1500 animals per 10 cm fog-2 RNAi plate. The process was repeated until the F7 generation, at which point the population was frozen for sequencing. The exact same process was performed for the N2 *fog-2(q71)* and XZ1516 *fog-2(q71)* to minimize the effects of being grown on different food sources.

**Sequencing and analysis**

Genomic DNA was prepared using Purelink Genomic DNA Mini Kit (invitrogen cat# K1820-01), libraries were constructed using the Nextera XT library kit (illumina cat# FC-131-1024), and sequenced on the NextSeq2000 platform. Demultiplexing was performed on basespace. FASTQ files were aligned to the WS276 genome assembly using *bwa mem* with default parameters (Li, 2014). GATK *ASEReadCounter* (v4.6.0.0) was used to count the number of reference and alternate alleles in each of the cross populations. The VCF from the 20220216 CaeNDR release was used to determine the segregating sites in the cross populations. The *xQTLStats* R package, which uses the methodology described in Huang et al., was used for calculating allele frequencies, standard errors, and *p* values associated with allele frequency differences between the crosses (Huang et al., 2020). Allele count tables generated by GATK *ASEReadCounter* were loaded into R using the *makeCountTables* function from the *xQTLStats* package. We next removed sites with fewer than 10 reads supporting the genotype calls and any sites that had zero counts for the reference or alternate alleles. We next ran the *calcAFD* function on the experimental allele count tables with the following parameters: sample.size=1e4, sel.strength=.95, bin.width=9000, eff.length=2000. Finally, we ran *calcContrastStats* to calculate the *p* values associated with the allele frequency differences between the RNAi and KO experiments. We submitted the sequencing reads to SRA with the BioProject number PRJNA1231493. We have provided the processed read counts and scripts to analyze them to the following GitHub repository: https://github.com/Thatguy027/fog2manuscript.

## Reagents

Strains:

**Table:** 

Strain	Allele name	Genotype	Description
N2			Wild *C. elegans* strain
XZ1516			Wild *C. elegans* strain
QX2538	*qq213*	*fog-2 ( qq213 [P17stop])* in XZ1516	XZ1516 *fog-2* knockout strain used to cross to QX2544
QX2544	*qq212*	*fog-2 ( qq212 [P17stop])* in N2	N2 *fog-2* knockout strain used to cross to QX2538

Bacteria:

**Table:** 

Strain	Allele name	Genotype	Description
*fog-2* RNAi		HT115 (DE3)[ *PL4440:: fog-2* ]	*fog-2* feeding bacteria for *C. elegans* feminization

## Data Availability

Description: Response to reviewer. Resource Type: Text. DOI: https://doi.org/10.22002/g9syn-e7837 Description: script to analyze allele counts. Resource Type: Workflow. DOI: https://doi.org/10.22002/cfp3q-gs478 Description: v1.0.0 of associated github repo. Resource Type: Software. DOI: https://doi.org/10.22002/yy2yv-s0n76
